# Ensemble Generation and the Influence of Protein Flexibility on Geometric Tunnel Prediction in Cytochrome P450 Enzymes

**DOI:** 10.1371/journal.pone.0099408

**Published:** 2014-06-23

**Authors:** Laura J. Kingsley, Markus A. Lill

**Affiliations:** Department of Medicinal Chemistry and Molecular Pharmacology, College of Pharmacy, Purdue University, West Lafayette, Indiana, United States of America; Duke University Medical Center, Duke University, United States of America

## Abstract

Computational prediction of ligand entry and egress paths in proteins has become an emerging topic in computational biology and has proven useful in fields such as protein engineering and drug design. Geometric tunnel prediction programs, such as Caver3.0 and MolAxis, are computationally efficient methods to identify potential ligand entry and egress routes in proteins. Although many geometric tunnel programs are designed to accommodate a single input structure, the increasingly recognized importance of protein flexibility in tunnel formation and behavior has led to the more widespread use of protein ensembles in tunnel prediction. However, there has not yet been an attempt to directly investigate the influence of ensemble size and composition on geometric tunnel prediction. In this study, we compared tunnels found in a single crystal structure to ensembles of various sizes generated using different methods on both the apo and holo forms of cytochrome P450 enzymes CYP119, CYP2C9, and CYP3A4. Several protein structure clustering methods were tested in an attempt to generate smaller ensembles that were capable of reproducing the data from larger ensembles. Ultimately, we found that by including members from both the apo and holo data sets, we could produce ensembles containing less than 15 members that were comparable to apo or holo ensembles containing over 100 members. Furthermore, we found that, in the absence of either apo or holo crystal structure data, pseudo-apo or –holo ensembles (e.g. adding ligand to apo protein throughout MD simulations) could be used to resemble the structural ensembles of the corresponding apo and holo ensembles, respectively. Our findings not only further highlight the importance of including protein flexibility in geometric tunnel prediction, but also suggest that smaller ensembles can be as capable as larger ensembles at capturing many of the protein motions important for tunnel prediction at a lower computational cost.

## Introduction

Over the past decade there has been a growing interest in a more detailed understanding of the ligand binding process. While resolving the lowest energy binding conformations of ligands in the binding site has long been a part of the drug design paradigm, there is an increasing need to understand the structural and kinetic process by which binding occurs. This can be especially complicated in proteins with deeply buried active sites where additional considerations must be made regarding the path, or tunnel, that the ligand uses to move from bulk solvent into the binding site. Although experimental methods, often in the form of mutational studies [1], [2], have offered some insight, they cannot provide systematic information about potential binding routes or detailed accounts of the ligand binding process. However, promising progress has been made toward these goals using computational methodologies [3], [4], [5], and such methods have become instrumental in generating experimentally testable hypotheses about the ligand binding process, especially in cases where the active site is buried [1], [5], [6].

Several computational approaches have emerged for predicting protein tunnels and providing detailed accounts of the physical properties of such tunnels. In general, these algorithms work by approximating the protein as a set of spheres and identifying continuous pockets of space that exist between these spheres. A grid is then constructed within these voids and each grid point is assigned a cost based on its proximity to surrounding protein atoms. Points which are close to the protein atoms are assigned a high cost, and points which are further away are assigned a lower cost. Then, tunnels connecting the buried active site to the surrounding bulk solvent with the lowest total cost are calculated based on this grid. Using this scheme, tunnels are ranked according to both the length and the narrowness of the tunnel, where the shorter and wider a tunnel is, the more favorably it is ranked. Variants of this method involve the use of Voronoi diagrams, different methods of representing the location of protein atoms, and different scoring/weighting schemes, however the basic premise is unchanged [7]. Such algorithms have been used to predict tunnels in a variety of enzymes [1], [2], [3] and channel/transporter proteins [5], [8].

Recent evidence has highlighted the importance of protein flexibility in protein access and egress tunnels [9], [10]. For instance, Stepankova *et. al.* demonstrated that at low concentrations organic solvents cause the widening of tunnels in haloalkane dehalogenase enzymes resulting in either increased activity or inhibition depending on the system and the solvent used [10]. Furthermore, in several cytochrome P450 (CYP) enzymes, phenylalanine clusters located in ligand entrance tunnels just above the active site act as gates that must be properly oriented to allow substrate binding [9], [11].

Ligand binding in CYP enzymes usually occurs on the order of milliseconds to minutes [12], and involves a series of conformational changes, some of which can be observed in as little as 10 nanoseconds [13], [14], [15] while others can take hundreds of nanoseconds or longer [16]. Thus, detailed investigations of the ligand binding process, such as those described above, often require the use of complex and resource intensive computational methods, such as random expulsion molecular dynamics [17]. These methods usually involve specialized molecular dynamics (MD) simulations of a particular ligand-tunnel pair and do not directly provide information about other possible tunnels or other potential ligands. Thus, geometric tunnel prediction methods remain a popular and widely used choice for initial, rapid analyses.

In many cases, geometric tunnel prediction in CYP enzymes is performed on a single crystal structure [18], [19], however there is a growing trend towards incorporating protein flexibility into tunnel prediction by using MD-based ensembles as is often done in docking studies [20]. Unlike in docking, where ensemble use has been extensively studied [21], in tunnel prediction, there have been no studies that directly investigate the influence of ensemble based prediction and there is little consensus about the number of ensemble members that are necessary. In tunnel prediction, ensembles have been generated by extracting frames at rates varying from one snapshot per nanosecond [19] up to 1000 snapshots per nanosecond [22]. Even minor secondary structure conformational changes in CYP enzymes may take tens or hundreds of nanoseconds of simulation time to observe [3], [12] which could result in ensembles containing several thousand members. The inclusion of several thousand snapshots can produce tens of thousands of tunnels, at which point the computational cost of prediction and analysis starts approaching that of the more advanced MD-based methods. Therefore, to remain efficient, ensembles used for geometric tunnel prediction must remain small, yet still incorporate important conformational changes.

The speed and ease of use provided by geometric tunnel prediction methods is highly dependent on the input necessary to obtain reasonable tunnel predictions. Yet, few, if any, studies have directly compared ensemble-based tunnel prediction to single structure tunnel prediction or investigated the different methods of ensemble generation and ensemble size in tunnel prediction. In this study, we used two prominent geometric tunnel prediction programs, Caver3.0 [22] and MolAxis [18], to predict tunnels in both static protein structures and MD-based ensembles of the apo and holo forms of three CYP isozymes (CYP119. CYP2C9, CYP3A4). The tunnels of these CYP enzymes have been well categorized [11] and thus provide an ideal system to study the effects of different ensemble generation methods on both the ability to predict known tunnels and the physical properties of those tunnels.

Differences in both the number of tunnels identified and physical characteristics of predicted tunnels were found depending on the structural input: crystal structure alone, apo ensemble, or holo ensemble. We tested a variety of clustering methods to generate small ensembles that retained the tunnel information present in larger ensembles. After attempting several clustering techniques, we found that none offered significant or consistent improvement over ensembles generated using time point-based selection. However, by combining time point-based selection ensembles from two different ensembles of the same protein, the apo and holo ensembles, we produced smaller ensembles that were comparable to larger ensembles in terms of identifying preferred tunnels. In addition, we found that pseudo-ensembles, derived from adding or removing a ligand from the crystal structure, also provide reasonable predictions of preferred tunnels. Our findings underscore the importance of protein flexibility in tunnel identification and also provide some general guidelines for geometric tunnel prediction on structural ensembles generated by MD simulations.

## Methods

### MD Simulations

High resolution CYP crystal structures where both the apo and holo forms were available were selected for this study; CYP119 (apo: 1IO9, resolution 2.05 Å and holo: 1F4T, resolution 1.93 Å), CYP2C9 (apo: 1OG2, resolution 2.60 Å and holo: 1OG5, resolution 2.55 Å), and CYP3A4 (apo: 4I3Q, resolution 2.60 Å and holo: 3UA1, resolution 2.15 Å). Pseudo-holo and -apo structures were generated by either adding the crystal ligand to the apo crystal structure or removing it from the holo crystal structure. The crystal ligand was added to the apo structure by aligning the apo and holo protein structures and combining the modified coordinates of the apo protein structure with the ligand structure from the holo form.

The heme parameters for a non-oxygenated state were extracted from the literature [23]. Reduce [24] was used to identify the proper rotamer and protonation states of histidine, and the proper rotamers of asparagine and glutamine side chains. Missing sidechains were added using the tleap module of Amber [25]. PyMOL [26]was used to rebuild the larger missing loop regions of CYP3A4. In the apo and holo forms of CYP3A4, residues 282–286 (KQTQS) and 280–286 (DSKQTQS), respectively, were rebuilt by hand using the PyMOL build tools. Residues were added sequentially and the structure was minimized, as described below, after the building process. In the holo form of CYP3A4, residues 259–261 (SRL) and 266–268 (KHR) were also missing, but this loop was present in the apo form, therefore we used the positions of these residues in the apo form as a guide to rebuild the loop in the holo structure. Missing terminal residues in any CYP structure were not rebuilt. Next, Gromacs-4.5.5 [27] was used to solvate each system in an octagonal water box of SPC216 waters and Na^+^ or Cl^−^ ions were added to neutralize each system. The box size was selected to guarantee a minimum distance of 10 Å between solute and box edge for the starting protein structure.

MD simulations were performed using Gromacs-4.5.5 [27] with the Amber03 force field [25]. For the holo structures, ligand charges and parameters were determined using the Antechamber [28] package from the Amber software suite, the GAFF force field and the AM1-BCC charge model. After solvation of the protein, 1000 steps of energy minimization were performed using the steepest descent method and particle mesh Ewald (PME) summation with a grid size of 0.12 nm and 4th order interpolation to compute potential, gradients and forces between the grid points. A switching function was applied to compute van der Waals interactions between 1.0 nm and the cut-off of 1.4 nm. The LINCS algorithm [29] was used to constrain bonds containing hydrogen atoms. The integration time step was 2 fs. Next, the hydrogen bond network of the surrounding waters was established using a 200 ps simulation in which all but the waters were restrained. This and all remaining simulations were performed at 300K using PME, Berendsen thermostat, and Parrinello-Rahman pressure coupling. A 300 ps equilibration run was performed to equilibrate all systems before the production run. This equilibration time was found to be suitable for all simulations, including the pseudo-simulations. The equilibration was followed by a 10 ns production MD run using the parameters described above.

### Ensemble Generation

#### Time Point Ensembles

Starting from the 10 ns MD trajectories, frames were extracted every 100 ps, 200 ps, 500 ps, 1000 ps, or 2000 ps to generate a total of five ensembles. The crystal structure and the minimized structure were added to each ensemble, generating ensembles of 103, 53, 23, 13 or 8 members (e.g. for the 2000 ps time point ensemble, frame 0, 2000, 4000, 6000, 8000, and 10000 were extracted from the trajectory and the crystal structure and minimized structure were added giving a final ensemble size of 8 members). The ensemble sizes selected are in line with those used in many ensemble docking studies [21], [30].We refer to this set of ensembles as the “time point ensembles”.

We compared time point based ensemble generation to ensemble generation based on three different clustering methods; RMSD, hydrogen bond network, and pairwise distance-based clustering. Both the crystal structure and the minimized structure were excluded from the clustering procedure. Using only the structures from the trajectory, or “reference” ensemble, we generated four sub-clusters equal in size to the smaller time point ensembles (e.g. 6, 11, 21, or 51 members). By using the structures of the 101-member ensemble as the input, the number of tunnels predicted in each subsequent ensemble could be calculated as a percentage of the total number of tunnels found in the 101-member reference ensemble. The different clustering methods and ensemble sizes could then be directly compared based on these percentages.

#### RMSD Ensembles

Clustering can be sensitive to noise caused by the highly flexible residues on the surface of the protein, thus these residues were not included in any of the clustering calculations used to generate ensembles. Surface exposed residues were calculated based on the crystal structure using the SwissPDBViewer [31], where the default settings were used.

To generate the RMSD ensemble, Gromacs was used to align the structures based on the C-alpha atoms, and the RMSD between all heavy atoms was calculated. Clustering of the structures was done using gromos clustering [27]. To generate ensembles containing a specified number of members, we iteratively increased the clustering cutoff. Once a specified cluster size was reached the clustering algorithm was terminated and tunnels were predicted in the resultant structures.

#### Pairwise-Distance

In addition to RMSD clustering, we clustered the structures based on distances between all heavy atom pairs. Unlike RMSD which compares the location of a given atom in two different structures, the pairwise-distance method compares the distances between given pairs of atoms in two structures.

After aligning all structures, the pairwise-distance similarity between each pair of structures, *k* and *n*, was calculated as:
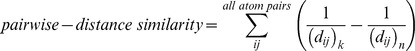
Where *ij* represents a pair of heavy atoms in the structure, and d*_ij_* is the Euclidian distance between those pairs. K-medoid clustering was used to cluster the structures based on the calculated similarity values.

#### Hydrogen Bond Network Ensembles

Hydrogen bonds present in each structure were calculated using the g_hbond module of Gromacs [27]. All possible pairs of structures were compared based on the hydrogen bond matrix generated by g_hbond. Each possible hydrogen bond position was evaluated, if both structures had the same hydrogen bond pattern at a given position (e.g. both had a hydrogen bond or both did not have a hydrogen bond), the position was assigned a 0, if they did not match a 1 was assigned. These values were averaged over the hydrogen bond matrix to produce a similarity score, where 0 represents an exact match of the hydrogen bond network and 1 represents complete dissimilarity. K-medoid clustering was used to cluster the structures based on the similarity score between the structures.

### Tunnel Calculation

Protein conformations of all ensembles were prepared for tunnel calculation by removing the hydrogen atoms, solvent, ions and ligand (if present). Tunnels were calculated in each structure using the programs MolAxis and Caver3.0. Both programs identify and report tunnels as a series of spheres with the radius of each sphere being defined as the distance to the nearest protein atom. The smallest sphere, which is the narrowest point along the tunnel, is denoted as the bottleneck and its radius as bottleneck radius. The tunnels are ranked according to a score, denoted flux value, that measures a combination of width and length of a tunnel. One major difference between MolAxis and Caver3.0 is that MolAxis pre-clusters tunnels prior to reporting, whereas Caver3.0 does not. This difference ultimately results in fewer, more distinct tunnels being reported by MolAxis and more, but in some cases, highly similar, tunnels being reported by Caver3.0. MolAxis found only a few tunnels with a bottleneck radius below 0.75 Å, therefore, to allow for a more equal comparison between the two programs we excluded tunnels that had bottlenecks below 0.75 Å. In Caver3.0, this was achieved by setting the probe radius to 0.75 Å, and in MolAxis, these tunnels were removed manually. All other settings for both Caver3.0 and MolAxis were left as default.

To initialize tunnel identification, a starting point inside the binding pocket was selected by using the iron atom of the heme as a reference point. The starting point was placed on the vector of the iron-sulfur bond 3 Å above the ligand-binding face of the heme for each protein conformation. In the case that one or both programs were unable to find tunnels from the initial starting point, the x, y, and z, coordinates were iteratively adjusted by adding or subtracting 1 Å from the initial starting point and the program was re-run until tunnels were identified. Tunnels that looped back into the protein or which had a bottleneck radius below the specified cutoff value were removed.

### Tunnel Clustering

After calculating tunnels in all members of each ensemble, we clustered the resultant tunnels to generate a representative set of tunnels for each ensemble. The RMS between each point in the tunnel and the nearest point (e.g. the minimum RMS) in the comparison tunnel was calculated. The overall RMSD was calculated by averaging over all of the individual RMS values for that tunnel pair. K-medoid clustering was used to cluster the tunnels and k was iteratively adjusted such that the maximum RMSD between any cluster member and the cluster center was less than 5.0 Å. The centroids of the resulting clusters represented the total number of tunnels found in a given ensemble.

The centroids were used to compare the tunnels found in each ensemble to the tunnels found in the reference ensemble (largest time point ensemble). Clusters from the comparison ensemble and the reference ensemble were considered to be the same tunnel if the RMSD between the centroids was less than the largest distance between the centroid and its original members. In other words, we ensured that the tested centroid would fit into the original cluster containing the reference centroid. This method was also used to perform comparisons across all ensembles.

All tunnels were identified and named using the descriptions and nomenclature for CYP tunnels set forth by Cojocaru *et. al.* [11]. Separate tunnels that are in close vicinity to one another are broken down into subclasses of a given tunnel; for instance tunnel 2a and 2ac, are known to be spatially nearby but are lined by different secondary structures. In addition to numbered tunnels, the “Solvent tunnel” (S) is a common feature in many CYP enzymes and is thought to control water access to the binding site [32].

## Results and Discussion

### Ensemble-based tunnel prediction vs. single structure tunnel prediction

**Figure 1:**
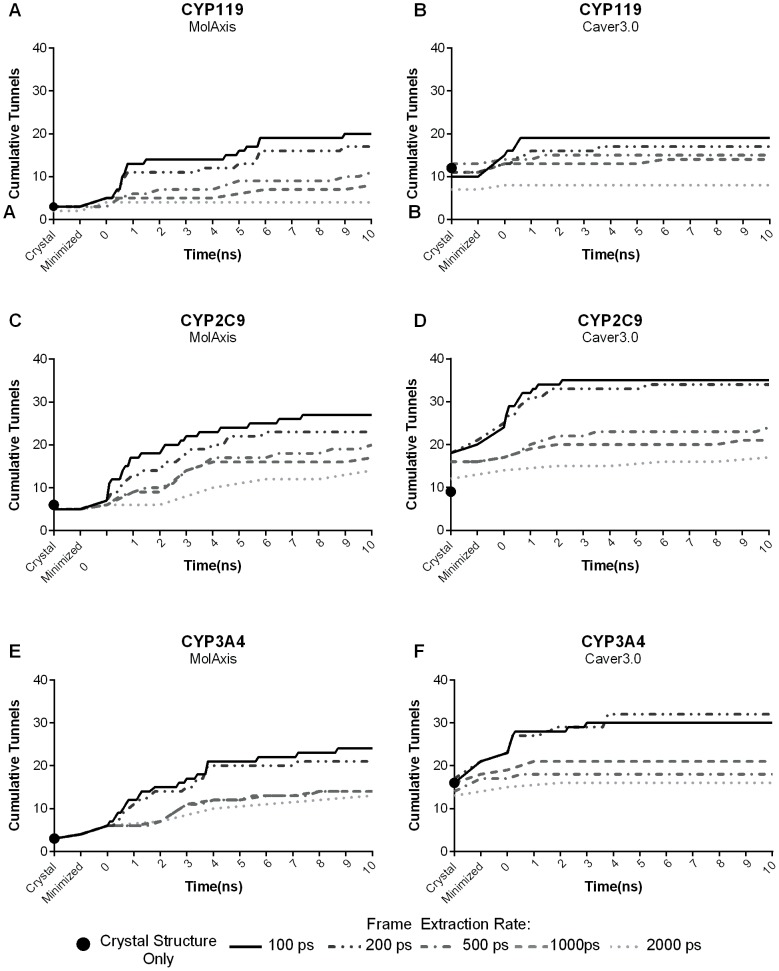
Cumulative number of tunnels found over time in the apo forms of CYP119 (A and B), CYP2C9 (C and D), and CYP3A4 (E and F) using the geometric prediction programs MolAxis (left column) and Caver3.0 (right column). Tunnels were predicted in five different ensembles, containing either 103, 53, 23, 13, or 8 members, generated by taking frames at evenly separated time points throughout the trajectory and adding the crystal and minimized structures. The tunnels were then clustered and the first appearance of each tunnel cluster was recorded. The black dot represents the number of tunnels in the crystal structure alone.

To assess the role that ensemble size plays in tunnel prediction, we compared the tunnels identified in the static crystal structure to those found in various structural ensembles. Ensembles of 103, 53, 23, 13, or 8 protein structures were generated and tunnels were predicted in each member using either Caver3.0 or MolAxis. The tunnels found in the ensembles were clustered and the cumulative number of tunnels identified over the length of the simulation is shown in Figure 1 (Figure 1 shows the apo ensembles only, the holo ensembles demonstrated a similar trend and are shown in Figure S1).

We first compared the tunnels identified in the raw crystal structure (black dots on Figure 1) to the total number of tunnels identified in the largest ensemble. Using MolAxis on the crystal structures produced no more than 20% of the total tunnels identified in the largest ensemble. Even using the smallest ensemble tested (8 members), MolAxis identifies twice as many tunnels as compared to the crystal structure alone. Caver3.0 slightly outperformed MolAxis, identifying on average about 50% of the total tunnels in the crystal structure. Tunnel prediction in the smallest ensemble using Caver3.0 also improved tunnel prediction, but not as drastically as was found with MolAxis.

Notably, using an ensemble can result in differences in the tunnels that are identified in each individual structure. This is most evident in the tunnels predicted by Caver3.0 in the crystal structure of CYP2C9. Clustering of the tunnels identified in the crystal structure alone resulted in a total of 9 tunnels. However, when the crystal structure was included as a member of either of the two largest ensembles, the number of tunnels found in the crystal structure doubled. Including additional tunnels that are on the outskirts of an original cluster can result in the formation of several smaller clusters derived from one larger cluster. In this case, several members of a large central cluster broke off to join nearby clusters of more closely related tunnels that only appeared in the trajectory. Whereas CYP2C9 showed the largest difference, in most cases the clustering inconsistencies resulted in a difference of only few tunnels, and overall, ensemble-based prediction in any size ensemble offered improvement over the crystal structure alone.

In general, the total number of tunnels identified increased with increasing ensemble size, but, in all cases, the greatest rate of new tunnel identification occurred during the first few nanoseconds of the simulation whereas few, if any, new tunnels were identified in the later stages. This trend was especially true for the two largest ensembles tested. Using Caver3.0, over 80% of the total tunnels were identified in the first nanosecond by the two largest ensembles. By the second nanosecond, there is a distinct plateau in the total number of tunnels identified in either ensemble (Figure 1 and S1, right column). A similar trend was observed using MolAxis, however the plateau was less distinct and occurred around 5–6 ns (Figure 1 and S1, left column).

The discrepancy between Caver3.0 and MolAxis in the rate at which tunnels were identified is likely due to algorithmic differences in the programs. In groups of nearby or highly similar tunnels, the MolAxis algorithm selects for tunnels with wider openings and neglects the more narrow tunnels in a process referred to as “overshadowing”. The removal of narrow, “overshadowed” tunnels is likely the reason that MolAxis consistently reports fewer tunnels per structure than Caver3.0, resulting in a slower plateau. When narrow tunnels are excluded by using a larger tunnel radius cutoff (1.25 Å cutoff), which is more representative of a biological ligands of CYP enzymes, we observed a drastic reduction in the total number of tunnels identified and better agreement in the rate of tunnel identification by MolAxis and Caver3.0 (Figures S2 and S3). While the overall number of tunnels identified decreased, the number of tunnels found in the crystal structure also decreased resulting in poorer overlap between the crystal structure and the largest ensemble.

One reason for the early spike in tunnel identification in the ensemble is the importance of small scale protein motions that can be captured in a relatively short time. Even slight conformational changes such as small rotamer state changes can drastically impact tunnel prediction results. In one instance, the phenylimidazole-bound form of CYP119 (Figure S1–A), five tunnels were found in the crystal structure, yet none met the 0.75 Å cutoff criteria resulting in 0% identification in the crystal structure. However, after minimization alone, the bottleneck of three of these tunnels expanded due to slight rotameric changes which allowed these three tunnels to be identified in the minimized structure.

The importance of small rotameric changes in tunnel prediction is further highlighted by comparing the apo and holo ensembles. In all tested data sets no more than 60% of tunnels were shared between the apo and holo ensembles. One of the main factors contributing to the observed differences is the steric restriction of binding site residues by the ligand.

**Figure 2:**
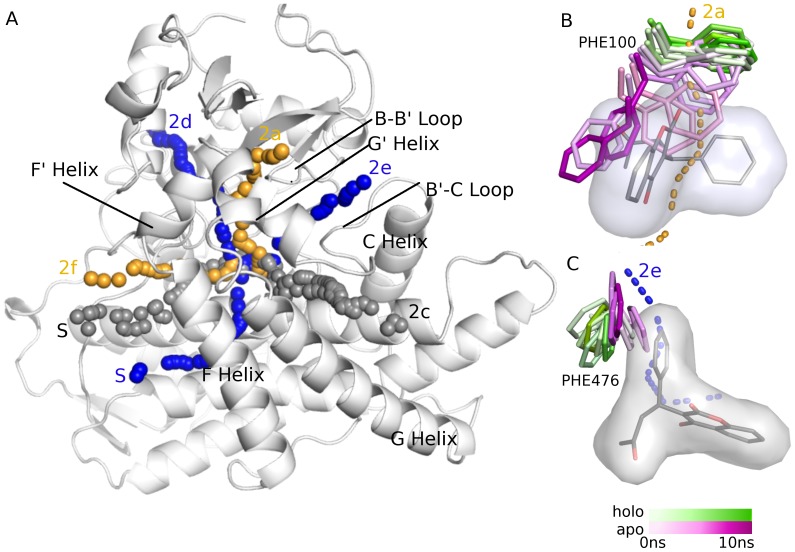
Differences in the largest tunnels predicted in the apo and holo ensembles of CYP2C9. **A**) Tunnels predicted in both the apo and holo ensembles using MolAxis are shown in grey, the tunnels found only in the holo ensemble are shown in blue (2d, 2e, and S) and the tunnels exclusively predicted in the apo form are shown in orange (2a and 2f). **B**) An example of how the presence of the ligand in the holo ensemble hinders identification of a tunnel that is present in the apo ensemble. In the apo ensemble (purple sticks) PHE100 rotates freely out of the 2a path whereas in the bound ensemble (green sticks) the presence of warfarin (grey sticks and surface) prevents rotation of PHE100 out of the 2a tunnel (orange dotted line). **C**) Example of how the absence of the ligand in the apo ensemble can interfere with tunnel identification. In the warfarin-bound ensemble (green sticks), steric interaction between warfarin (grey sticks and surface) and PHE476 prevents the residue from closing off tunnel 2d (blue dashed line), while in the apo ensemble (purple sticks) the absence of warfarin allows PHE476 to constrict the bottleneck of the tunnel and thus tunnel 2d is not identified in the apo ensemble.

Figure 2A shows the differences in the top-3 widest tunnels identified in the holo (blue tunnels) and apo (orange tunnels) ensembles of CYP2C9 (in the holo ensemble 2c and 2f both had the same bottleneck radius, thus four tunnels were counted in the top 3). All of the tunnels (2a, 2d, and 2e) that differ between the two ensembles pass directly through residues of the warfarin binding site, just below the FG helix block. The absence of warfarin in the apo ensemble allows for free rotation of PHE100 resulting in the opening of tunnel 2a (Figure 2B). However, the presence of warfarin is required for the opening of tunnels 2d and 2e. The restriction of PHE476 by warfarin allows for the opening of tunnel 2d (Figure 2C), while the restriction of PHE114 allows for the formation of tunnel 2e (not shown). The allowed rotamer states of these residues in their respective ensembles caused a preferential opening of certain tunnels and the closure of others. The degree of this preference is highlighted by the ranking of these tunnels in the apo versus the holo ensemble. For instance, even though tunnel 2a was found in both the apo and holo ensemble of CYP2C9, in the apo ensemble, it was ranked 1^st^ according to the flux value and had a bottleneck radius of 1.64 Å, while in the holo ensemble it was ranked 19^th^ and had a bottleneck radius of 0.93 Å, according to MolAxis. This was a consistent trend for tunnels 2d and 2e using both Caver3.0 and MolAxis. Interestingly, the two tunnels that were found in common in the apo and holo ensembles, 2c and the solvent tunnel S (grey paths), do not directly interact with residues of the warfarin binding site.

Minor conformational changes can drastically influence tunnel prediction in even the widest identified tunnels and most of these changes can be captured in the early stages of a simulation. While any sized time point ensemble offered improvement over the crystal structure alone, in order to produce the most detailed tunnel prediction results ensembles containing over 50 members were required. In these ensembles we found that the majority of tunnels were identified within the first several nanoseconds of a trajectory. In other words, the bulk of tunnels can be found in a small set of randomly selected structures, but the remaining tunnels are contributed by specific structural members that might be sampled later in the MD simulation. We hypothesized that by identifying the specific members that contributed unique tunnels and combining those with a few other structures that we could produce smaller ensembles that performed as well as ensembles containing over 100 members.

### Ensemble Generation using Clustering

Using time point based ensembles is a straightforward method, frequently used to generate ensembles in docking [21] and to a lesser extent in tunnel prediction [22], [33]. However, this method relies on heavy sampling to cover the conformational space of the protein and therefore typically requires a large number of structures. The inclusion of a large number of structures in an ensemble for tunnel prediction can become computationally costly in terms of both time and memory [22]. Thus we attempted to generate smaller, more productive ensembles for tunnel prediction by utilizing clustering techniques. We hypothesized that highly similar protein conformations would produce similar tunnels while dissimilar structures would produce different tunnels. We used three methods of clustering to generate the ensembles: heavy atom RMSD based, pairwise-distance based, and hydrogen-bond network based clustering.

Heavy atom RMSD based clustering is used routinely to generate ensembles for various computational techniques such as ensemble docking. Akin to RMSD based clustering, pairwise-distance based clustering compares changes in the distances between pairs of atoms in the protein. Hydrogen bonding is known to play a role in tunnel closing/opening to allow ligand entry and egress [11], [34] and therefore we also generated ensembles based on conserved hydrogen bonding patterns. In order to reduce the potentially dominant RMSD contribution of highly flexible surface exposed residues, which are not likely to be critical determinants of internal tunnels, these residues were excluded in all cases.

**Figure 3:**
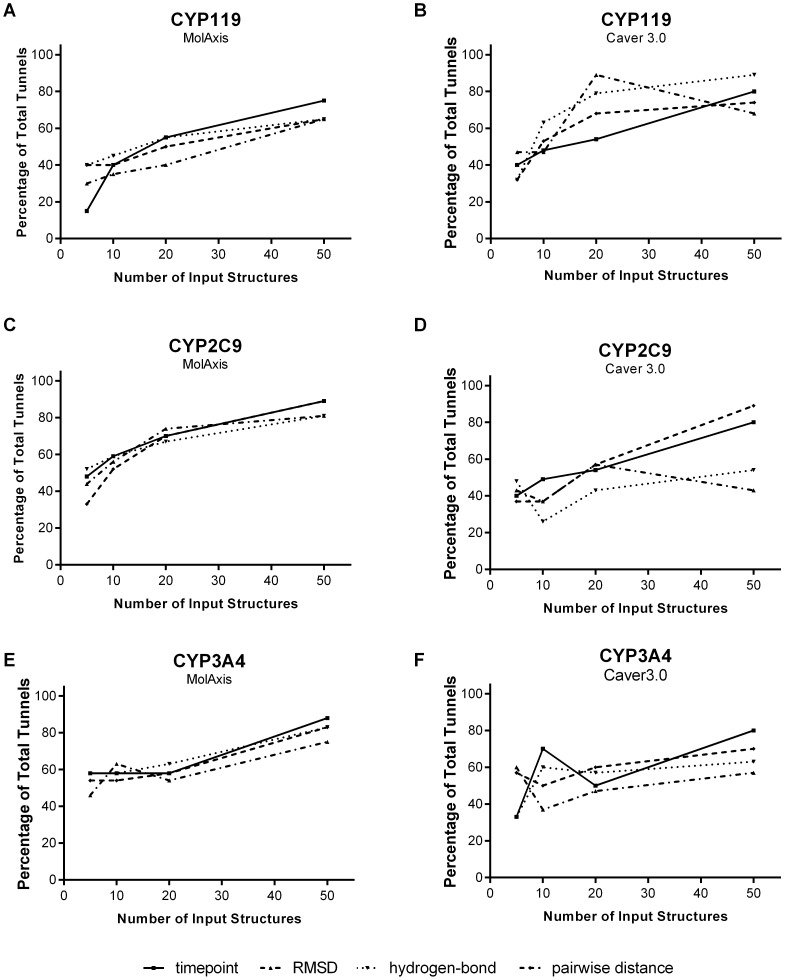
Percentage of the total reference tunnels identified using time point ensembles, RMSD-clustered ensembles, hydrogen bond network ensembles, and pairwise-distance ensembles in the apo form of CYP119 (A and B), CYP2C9 (C and D) and CYP3A4 (E and F) using MolAxis and Caver3.0.

Using these clustering techniques we generated ensembles from the MD simulations containing either 6-, 11-, 21- or 51-members and compared the tunnels identified by these ensembles to the time point ensemble of the same size (the crystal and minimized structures were excluded from all ensembles). We found, on average, that none of the clustering methods tested produced observable enrichment in the number of tunnels identified compared to the time point ensembles of the same size (apo results are shown in Figure 3 and holo results are shown in Figure S4). We believe that this is due to the sensitivity of geometric prediction methods to minor changes in protein structure that cannot be adequately accounted for by a single global descriptor. For instance, the slight rotation of a side chain may have a low impact on the structural RMSD, but could cause severe restriction of a given tunnel.

While no method of ensemble production consistently out- or under-performed other methods, all methods shared some degree of tunnel loss due to clustering. Tunnel loss occurs when fewer tunnels are found in a larger ensemble then in the previous smaller ensemble, for instance in CYP3A4, using Caver3.0 fewer tunnels are found in the 21- member time point ensemble than in the 11-member time point ensemble. Information loss can occur between two ensembles by the inclusion of additional tunnels that lie in boundary regions between two existing clusters. In some cases this can lead to the merging of two smaller clusters into a single larger cluster resulting in a decrease in the total number of tunnels identified. The implications of tunnel loss due to tunnel clustering are discussed further in the “Shortcomings of Ensemble Based Tunnel Prediction” section.

In addition to clustering of the tunnels themselves, clustering the input protein structures proved difficult. One reason for this may be that clustering structures to optimize tunnel identification may require more precise input. In docking studies this is often achieved by including only residues from the binding site in clustering. In order to use standard clustering techniques for tunnel prediction, it may be necessary to not only remove the noise created by surface exposed residues but to restrict clustering to only those residues that line a specific tunnel of interest. However, unlike in docking where the binding site is usually known *a priori*, this information would not be known prior to performing the tunnel prediction calculations and thus would not likely be of practical use for tunnel identification. The difficulties of clustering-based ensemble generation make it an unlikely choice to reduce the ensemble size necessary for geometric tunnel prediction. While our findings indicate that larger ensembles are necessary to produce the most complete tunnel predictions, we assessed whether larger ensembles were also necessary to extract the most relevant tunnel information.

### Identification of Preferred Tunnels

Ligand egress is difficult to study directly using experimental methods, however, using resource-intensive computational approaches, many mammalian CYP enzymes, including CYP2C9 and CYP3A4, have been heavily studied [34], [35], [36]. In both CYP2C9 and CYP3A4 several tunnels have been identified as favorable for ligand passage using advanced MD methods, these tunnels are thought to be “preferred” exit tunnels for several ligands [37]. Using the largest time point ensembles, we identified where along the trajectory these preferred tunnels occurred and assessed their topological features as well as the persistence of the tunnels over the length of the trajectory.

**Figure 4:**
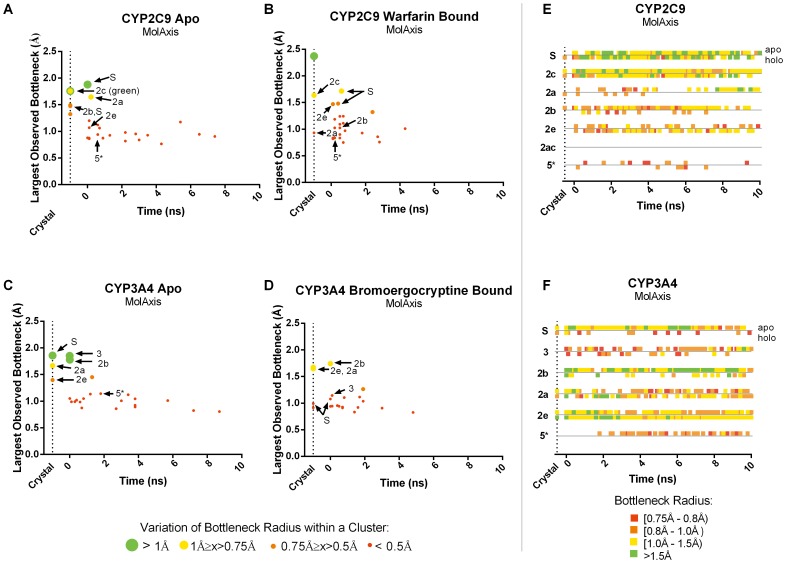
Occurrence and bottleneck widths of preferred tunnels predicted by MolAxis in CYP2C9 and CYP3A4. (**A–D**) The time point at which each tunnel first observed is plotted against the widest bottleneck radius observed, at any point, for that tunnel. The color and size of the dot corresponds to the average fluctuation in the bottleneck of that tunnel over time, tunnels which frequently oscillate between wide open and mostly closed are shown in large green dots, alternatively those tunnels in which the bottleneck is relatively inflexible, retaining a more or less constant diameter are shown in red dots. Preferred tunnels are denoted with arrows. Furthermore, the bottleneck radii of preferred tunnels over time are shown in **E** and **F.** Green represents a more open bottleneck, where red represents a more closed bottleneck. ^*^Tunnel 5, is not a preferred tunnel, but rather a “rare” tunnel that serves as a point of reference to evaluate the preferred tunnels.

**Figure 5:**
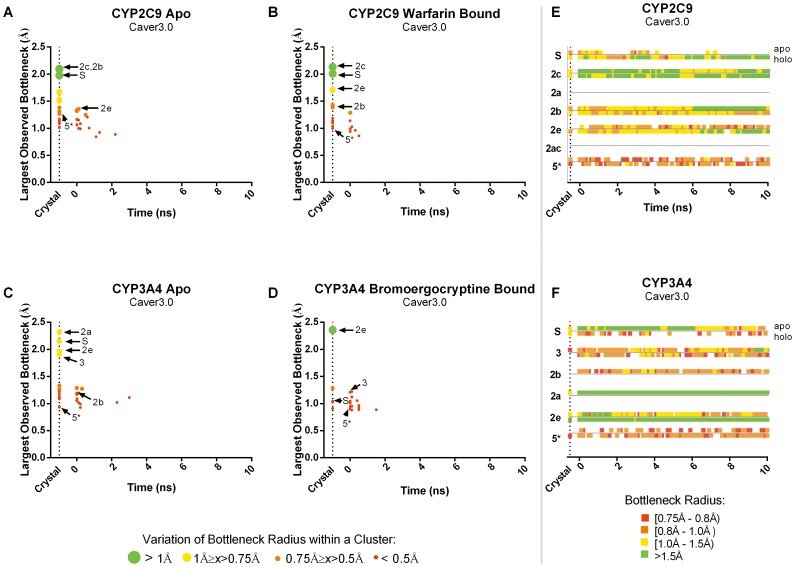
Occurrence and bottleneck widths of preferred tunnels predicted by Caver3.0 in CYP2C9 and CYP3A4. (**A–D**) The time point at which each tunnel first observed is plotted against the widest bottleneck radius observed (at any point) for that tunnel. The color and size of the dot corresponds to the average fluctuation in the bottleneck of that tunnel over time. Preferred tunnels are denoted with arrows. Furthermore, the bottleneck radii of biologically preferred tunnels over time are shown in **E** and **F.** Green represents a more open bottleneck, whereas red represents a more closed bottleneck. ^*^Tunnel 5, is not a preferred tunnel, but rather a “rare” tunnel that serves as a point of reference to evaluate the preferred tunnels.

For all tunnels identified, we plotted the time point at which the tunnel was first observed against the largest bottleneck radius observed in that tunnel cluster (Figures 4 A–D and 5 A–D). Preferred tunnels 2a, 2b, 2c, 2e and solvent (S) for CYP2C9 and 2a, 2b, 2e, 3 and S for CYP3A4 are denoted with an arrow for each system. Preferred tunnel 2ac of CYP2C9 was not found therefore is not indicated in Figures 4 and 5. The bottleneck radius of the tunnel over the duration of the trajectory is also shown in Figures 4 and 5 (E and F).

In general, preferred tunnels tended to be among those tunnels with the largest observed bottleneck radii and showed the largest fluctuations in bottleneck radii. For comparison, a non-preferred tunnel, tunnel 5, is also shown. Tunnel 5 is known as “rare” tunnel and it has not yet been implicated in biological function or ligand egress [34]. This tunnel is spatially distinct from preferred tunnels and demonstrates different behavior than the preferred channels. In both MolAxis and Caver3.0 the tunnel is found to have only minor fluctuations in the bottleneck radius and remains closed or partially closed throughout the trajectory. This is in stark contrast to the preferred tunnels, some of which were found to have deviations exceeding 1.5 Å between the largest and smallest observed radii for a single tunnel. This finding is consistent with the idea that bottlenecks regions are highly flexible and may act as gates to allow/deny ligand passage. Fluctuations of this magnitude could prevent identification of even large tunnels in certain static structures.

In only one case, the holo form of CYP2C9 using Caver3.0, the crystal structure alone accounted for all of the preferred tunnels that were identified in that trajectory. However, in all other systems, preferred tunnels that were not identified in the crystal structure, were identified very early in the trajectory and in most cases, were consistently identified throughout the duration of the trajectory. In general, most, but not all of the preferred tunnels were identified in the smallest (7-members) ensembles (Table 1).

**Table 1:** Percentage of biologically preferred tunnels identified in ensembles.

	MolAxis				Caver3.0			
	Crystal Structure	7- member parent ensemble	14-member mixed ensemble^a^	103- member ensemble	Crystal Structure	7- member parent ensemble	14-member mixed ensemble^a^	103- member ensemble
CYP2C9 apo	67%	67%	83%	83%	50%	67%	67%	67%
CYP2C9 holo	33%	67%		67%	67%	67%		67%
CYP3A4 apo	60%	100%	100%	100%	80%	80%	40%	100%
CYP3A4 holo	60%	60%		100%	20%	60%		60%

a The mixed ensemble contains the 7-member parent ensembles from both the apo and holo simulations.

While the use of an ensemble improved tunnel prediction results in both the full data set and in the preferred tunnels only data set, the inclusion of the ensemble alone does not guarantee comprehensive tunnel prediction. Some tunnels were found to have very different physical properties in the apo ensemble versus the holo ensemble. In the most extreme case, tunnel 2a in CYP3A4 was found in the apo, but not the holo ensemble by Caver3.0. These differences suggest that a consensus-based approach which combines tunnel prediction results from both the holo and apo forms may provide improved predictions. To the best of our knowledge, no studies have been performed that compare tunnel prediction in apo/holo mixed ensembles.

### Mixed Ensemble Tunnel Prediction

**Figure 6:**
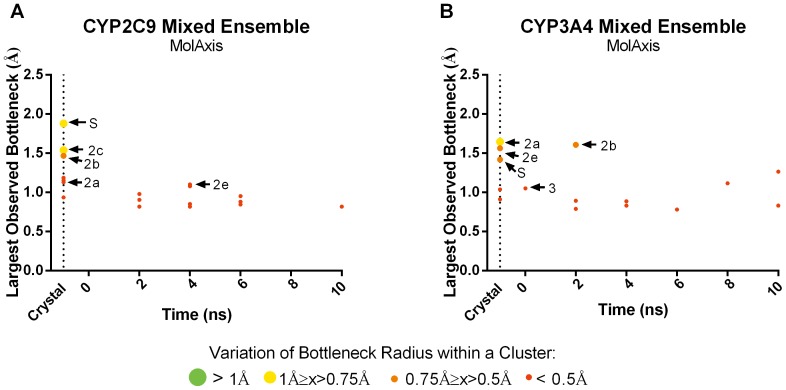
Occurrence and bottleneck widths of preferred tunnels predicted by MolAxis in 14- member apo/holo mixed ensembles in CYP2C9 (A) and CYP3A4 (B). The time point at which each tunnel is first observed is plotted against the widest bottleneck radius observed (at any point) for that tunnel. The color and size of the dot corresponds to the average fluctuation in the bottleneck of that tunnel over time.

**Figure 7:**
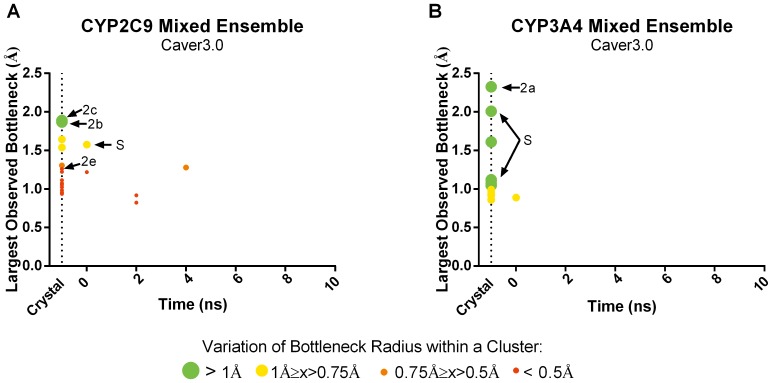
Occurrence and bottleneck widths of preferred tunnels predicted by Caver3.0 in 14- member apo/holo mixed ensembles in CYP2C9 (A) and CYP3A4 (B). The time point at which each tunnel first observed is plotted against the widest bottleneck radius observed (at any point) for that tunnel. The color and size of the dot corresponds to the average fluctuation in the bottleneck of that tunnel over time.

A 14-member ensemble was generated by combining seven snapshots (the crystal structure plus frames taken every 2 ns) from the holo and apo trajectories for both CYP2C9 and CYP3A4. Caver3.0 and MolAxis were run on each member of these apo/holo mixed ensembles and the resultant tunnels were clustered. For all tunnels identified, we again plotted the time point at which the tunnel was first observed against the largest bottleneck radius observed in that tunnel cluster (Figure 6 and Figure 7). Tunnel prediction in the 14-member mixed ensemble was compared to both the apo and holo parent (7-member) ensembles and to the largest (103 members) apo and holo ensembles tested.

By combining the apo and holo ensembles, in three out of four cases, we were able to identify the same number of tunnels or even more tunnels compared to using either the apo or holo 103- member ensembles alone (Table 1). The smaller mixed ensembles contained fewer tunnels overall while still preserving all of the preferred tunnels resulting in reduced noise in the prediction data. Furthermore, using the smaller ensembles improved the rate at which preferred tunnels were identified. For instance, tunnel 2e was found in the holo crystal structure, but not the apo crystal structure of CYP2C9 and by using the mixed ensemble, this tunnel was predicted earlier than it would have been in the apo ensemble alone. As with the non-mixed ensembles, the bottleneck radii of the preferred tunnels in the mixed ensembles were usually among the largest and most flexible identified. Using MD to take advantage of different conformational states of each unique crystal structure input, smaller apo/holo mixed ensembles outperform tunnel prediction in the parent crystal structures and were comparable to non-mixed ensembles containing over 100 members in most cases.

### Pseudo-Ensemble Tunnel Prediction

Although the performance of the mixed ensemble was encouraging, lack of structural data may preclude the use of this method in enzymes where only a single structure, either apo or holo, is available. One way to address this problem is to either remove the co-crystallized ligand from the holo structure, resulting in a pseudo-apo structure or to add an exogenous ligand to the apo structure resulting in a pseudo-holo structure. Although such techniques are common in *in silico* studies, such as docking, this technique has not yet been used in tunnel prediction. We produced a series of pseudo-ensembles and assessed whether the tunnels predicted in those pseudo-ensembles provided an accurate estimate of the tunnels predicted in the true-apo or true-holo ensembles.

First, pseudo-apo and pseudo-holo structures of both CYP2C9 and CYP3A4 were generated by removing the ligands, warfarin and bromoergocryptine, respectively, from the holo structures or adding these ligands to the apo structures. A 10 ns MD simulation was performed on each of these pseudo-structures and “pseudo-ensembles” were generated by extracting frames every 100 ps, as previously described. The tunnels predicted in pseudo-apo and pseudo-holo ensembles were compared to those predicted in the true-apo and true-holo ensembles, respectively. The prediction accuracy was calculated as the percentage of true-apo (or true-holo) tunnels identified by the pseudo-apo (or pseudo-holo) simulation. As a point of comparison, the percentage of shared tunnels in the original, unaltered simulation was also calculated (this value has been denoted in parenthesis in the “prediction accuracy column”). For instance, because both the pseudo-apo and the true-holo simulations start from the same crystal structure, we compared the tunnels in both of these ensembles to those in true apo-ensemble to determine whether the removal of the ligand itself was responsible for differences in tunnel identification between the structures.

Both the pseudo-apo and pseudo-holo ensembles typically captured around 65% of the tunnels identified in the true-apo or true-holo ensemble (Table 2). The pseudo-apo simulations resulted in a more consistent and greater overall increase, compared to the original simulation, in the percentage of accurately predicted tunnels. This was especially true in the case of CYP2C9, where the pseudo-apo simulation resulted in a nearly 20% increase over the holo simulation using MolAxis and an increase of nearly 30% using Caver3.0.

**Table 2:** Tunnel prediction using pseudo-ensembles.

	MolAxis				Caver3.0			
	Prediction Accuracy (original ensemble)^a^	7- member parent ensemble	14-member pseudo-mixed ensemble^b^	103- member ensemble	Prediction Accuracy (original ensemble)^a^	7- member parent ensemble	14-member pseudo-mixed ensemble^b^	103- member ensemble
CYP2C9 Pseudo-apo	70% (52%)	67%	83%	83%	76% (49%)	50%	67%	67%
CYP2C9 Pseudo-holo	65% (62%)	67%	83%	83%	57% (85%)	50%	67%	50%
CYP3A4 Pseudo-apo	67% (58%)	100%	100%	100%	67% (60%)	80%	40%	80%
CYP3A4 Pseudo-holo	52% (62%)	60%	80%	80%	76% (68%)	80%	80%	80%

a The original ensemble is the unaltered ensemble, from which the input crystal structure for the pseudo-ensemble was taken. For example in the case of the pseudo-apo ensemble the original ensemble is the true-holo ensemble because both were derived from the 1OG5 crystal structure.

b The pseudo-mixed ensemble contains both the pseudo- and true-simulation derived from the same crystal structure. For instance, the, CYP2C9 14-member pseudo-mixed ensemble consists of 7 members from both the CYP2C9 pseudo-apo simulation and the true holo simulation.

Using the pseudo-simulations, we also constructed pseudo-mixed ensembles and calculated the percentage of preferred tunnels identified in these ensembles as compared to the true-mixed ensembles (Table 2). For instance, seven members each from the pseudo-apo and true-holo simulations were combined into a single pseudo-mixed ensemble. In all but one case, the pseudo-mixed ensembles performed as well as the true mixed ensembles at predicting preferred tunnels. Notably, in the one case that deviated from this trend, the prediction accuracy was the lowest of any of pseudo-simulation tested (pseudo-holo ensemble of CYP2C9 predicted by MolAxis).

**Figure 8:**
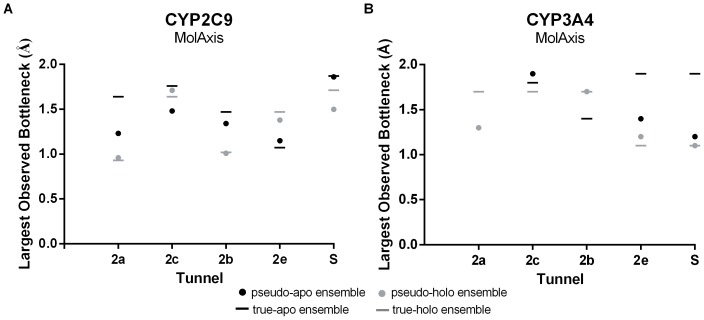
A comparison of the bottleneck radii in the preferred tunnels reported by MolAxis in the true- and pseudo- ensembles for both CYP2C9 (A) and CYP3A4 (B). The largest observed bottleneck for each preferred tunnel is shown for all simulations; the pseudo-apo and true-apo are shown in black, as dots and lines, respectively, and the pseudo-holo and true-holo simulations are shown in grey using the same symbols. Although the pseudo-simulations start from the holo/apo crystal structure, once the ligand is removed/added, to generate the pseudo-simulation, in many cases, the bottleneck radii starts approaching that of the true-apo/true-holo simulation (e.g. the black dots approach the black bars and the grey dots approach the grey bars).

Ideally, both the number and physical properties of the tunnels from the pseudo-simulations should more closely resemble the tunnels of the true simulation than those of the original simulation. For each of the preferred tunnels, we compared the largest bottleneck radius observed in the true-simulations to the largest observed bottleneck radius in the pseudo-simulations (Figure 8 and Figure S5). In many, but not all, cases we found that tunnel bottlenecks in the pseudo-simulations started approaching those in the true simulations. For instance, the bottleneck in tunnel 2a of CYP2C9 was found to increase from 0.93 Å in the true-holo simulation to 1.23 Å in the pseudo-apo simulation, more closely resembling the bottleneck in the true-apo simulation (1.64 Å). Likewise in the pseudo-holo simulation, the bottleneck decreased from 1.64 in the true-apo to 0.96 Å in the pseudo-holo simulation, more closely resembling the true-holo simulation (0.93 Å).

**Figure 9:**
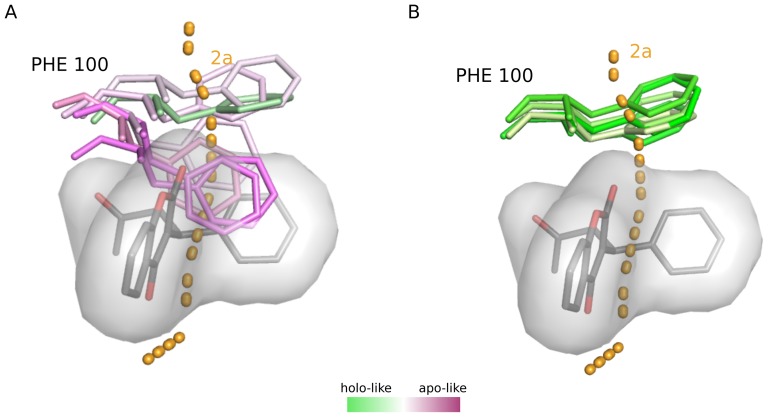
Rotameric states of PHE100 in the pseudo-simulations of CYP2C9. **A**) In the pseudo-apo simulation, PHE100 initially resembles its position in the true-holo simulation (green sticks) where it does not sterically interfere with the ligand. However, throughout the course of the simulation, PHE100 rotates and more closely resembles its conformation in the true-apo simulation, entering space that was originally occupied by the ligand. **B**) In the pseudo-holo simulation, the addition of the ligand forces PHE100 to adopt a conformation that is similar to its conformation in the true-holo simulation.

The removal or addition of the ligand in the pseudo-simulations allows for binding site residues in close proximity to the ligand to adopt a unique ensemble of conformations containing rotamers similar to those in both the true-apo and true-holo ensemble. For instance in tunnel 2a of CYP2C9, the rotameric states of PHE100 in the pseudo-apo simulation initially resemble the true holo-simulation (Figure 9A, green sticks), but quickly progress towards conformations that are only observed in the true-apo simulation (Figure 9A, purple sticks). Although, in the pseudo-apo simulation PHE100 did not fully capture all rotameric states that were observed in the true-apo simulation (see Figure 2B, dark purple sticks), the expanded motion allowed for a significant increase in the bottleneck radius as shown in Figure 8A. Likewise, when the ligand was added to the apo conformation, PHE100 becomes highly restricted (Figure 9B), as is observed in the true-holo simulation, resulting in a significant decrease in the bottleneck radius of tunnel 2a (Figure 8A).

Taken together, this data suggest that, in the case of lack of structural data, pseudo-simulations may provide a viable alternative. We observed that a majority of the preferred tunnels were identified in both pseudo-ensembles and in addition found that, in many cases, the bottleneck radius of the tunnels identified in the pseudo-simulations either increased or decreased to more closely resemble the true-simulations.

### Shortcomings of Ensemble Based Tunnel Prediction

In general we found ensemble based approaches, specifically mixed ensembles, to be advantageous for geometric tunnel prediction; however, the inclusion of multiple protein structures in geometric tunnel prediction heavily relies on clustering of the resultant tunnels. Clustering is necessary to analyze the multitude of tunnels produced from an ensemble, but can also directly influence the outcome of tunnel prediction. The type of clustering used, the number of input structures, and the cutoff parameters selected can all affect the results of geometric tunnel prediction and in some cases can negatively impact the outcome.

For instance, loss of data due to clustering the Caver3.0 tunnels in the mixed ensemble of CYP3A4 leads to the poor performance of the mixed ensemble compared to non-mixed ensembles of any size. The poor performance of the mixed ensemble was the result of a larger central cluster, identified as tunnel 2a, which merged with two nearby clusters, identified as tunnels 2b and 3. The centroid of the tunnel 2a cluster was found to be extremely short, a fairly frequent occurrence in the tunnels predicted by Caver3.0. Comparing a tunnel to an exceedingly short, nearby tunnel can artificially lower the RMSD between the two members because the initial portions of the tunnels will match very closely. This was true for both the apo and the mixed ensembles, however, in the large apo ensemble, divergent members of clusters 2b and 3 were found that were further separated from the 2a centroid. This variety allowed for new clusters (e.g. 2b and 3) to be formed in the apo ensemble. These clusters were not formed in the mixed ensemble because the overall number of tunnels was smaller as were the number of divergent members that would result in new cluster formation.

Tunnel loss of this type due to clustering could have severe implications for smaller tunnels that lie between two larger tunnels. Tunnel 2ac, a preferred tunnel in CYP2C9, exits between the BC loop and the G helix, and lies between tunnels 2a and 2c. This tunnel was not found in any CYP2C9 trajectory using either method. Tunnel 2ac is known to merge with nearby channels during simulations [11], which is likely a contributing factor to why this tunnel was overlooked using both methods.

Clustering may not only introduce the risk of overlooking smaller, or nearby tunnels, but the three dimensional location alone may not fully reflect the specific details of a given tunnel. For instance the identity and the conformations of the tunnel lining residues are not taken into account. Two nearby tunnels may have completely different physicochemical properties that would not be captured by location-based clustering alone. Including such features may provide an improvement on the currently used distance based techniques, although to date, there have not been widespread attempts to include the physicochemical properties, or tunnel lining residues of a given tunnel into the clustering procedure. Ensemble-based tunnel prediction and tunnel clustering are inextricably linked. However due to the sensitivity of clustering, careful consideration must be used to achieve a balance between preventing redundancy and preserving critical tunnel features.

## Conclusions

One of the main challenges in tunnel prediction is the incorporation of protein flexibility in an efficient manner. We used MD simulations to generate a series of ensembles and compared tunnel prediction results in ensembles of various sizes and compositions to the tunnels predicted in a single static crystal structure. We found that there was a sharp increase in the number of tunnels identified within the first several nanoseconds of the MD trajectory and including as few as seven members could capture these additional tunnels and improve tunnel prediction results when compared to the crystal structure alone We introduced a novel ensemble generation method that combines time point based snapshots from both an apo and a holo trajectory for a given CYP system and found that, in three out of four cases, this method performs as well or better than significantly larger ensembles generated from the apo or holo trajectory alone. Additionally, we found that, in the absence of structural data, pseudo-ensembles may be suitable for use in tunnel prediction.

While MD simulations are the most typical source of structural ensembles for geometric tunnel prediction, time constraints often limit such simulations to small scale motions. We found that these small scale motions accounted for the opening and closing of several tunnels throughout the duration of each simulation and resulted in, at times, drastic fluctuations in the bottleneck radii of tunnels belonging to a single cluster. Interestingly, preferred tunnels were often found to have the largest bottlenecks and also the most variation in bottleneck radius. This finding is in line with previous findings that bottleneck residues may act as gates to dictate access of various substrates to the binding site [36], [38].

Although in this study we focused on three enzymes of the CYP family, the properties of the three binding sites differ quite significantly. Thus, we believe that these findings are applicable at least to the broader CYP family as well as other similar enzymes with multiple tunnels and buried active sites. Taken together, our findings highlight the importance of both ensemble generation and selection in the tunnel prediction process, but also demonstrate that exceedingly large ensembles do not necessarily provide an advantage over smaller ensembles.

A more holistic knowledge of the ligand binding process has become increasingly important in recent years as we have begun to realize some limitations of traditional computational techniques. Tunnel prediction is emerging as a useful computational tool to address questions involving ligand entrance and egress and there has been growing interest in building and improving tools in this field. To date, tunnel prediction has proven useful in fields ranging from understanding chemical mechanisms in channels [38], to protein engineering [6] and drug design [39] and a better understanding of the most efficient and practical ways to calculate tunnels has great potential to contribute to this growing field.

## Supporting Information

Figure S1 Cumulative number of tunnels found over time in the apo forms of CYP119 (**A and B**), CYP2C9 (**C and D**), and CYP3A4 (**E and F**) using the geometric prediction programs MolAxis (left column) and Caver3.0 (right column). Tunnels were predicted in five different ensembles, containing either 103, 53, 23, 13, or 8 members, generated by taking frames at evenly separated time points throughout the trajectory and adding the crystal and minimized structures. The tunnels were then clustered and the first appearance of each tunnel cluster was recorded. The black dot represents the number of tunnels in the crystal structure alone.
(TIF)

Figure S2 Tunnel prediction as a function of sampling time and ensemble size in the apo ensembles (1.25 Å cutoff). Cumulative number of tunnels found over time using a bottleneck cutoff of 1.25 Å in the apo forms of CYP119 (**A** and **B**), CYP2C9 (**C** and **D**), and CYP3A4 (**E** and **F**) using the static prediction programs, MolAxis (left column) and Caver3.0 (right column). Tunnels were predicted in five different ensembles, containing either 103, 53, 23, 13, or 8 members, which were generated by taking frames at specific time points throughout the trajectory and adding the crystal and minimized structures.
(TIF)

Figure S3 Tunnel prediction as a function of sampling time and ensemble size in the holo ensembles (1.25 Å cutoff). Cumulative number of tunnels found over time using a bottleneck cutoff of 1.25 Å in the holo forms of CYP 119 (**A** and **B**), CYP2C9 (**C** and **D**), and CYP3A4 (**E** and **F**) using the static prediction programs, MolAxis (left column) and Caver3.0 (right column). Tunnels were predicted in five different ensembles, containing either 102, 52, 22, 12, or 7 members, which were generated by taking frames at specific time points throughout the trajectory and adding the crystal and minimized structures.
(TIF)

Figure S4 Percentage of the total reference tunnels identified using time point ensembles, RMSD-clustered ensembles, hydrogen-bond network ensembles, and pairwise-distance ensembles in the holo form of CYP119 (**A** and **B**), CYP2C9 (**C** and **D**) and CYP3A4 (**E** and **F**) using MolAxis and Caver3.0.
(TIF)

Figure S5 A comparison of the bottleneck radii in the preferred tunnels reported by Caver3.0 in the true- and pseudo- ensembles of CYP2C9 (**A**) and CYP3A4 (**B**). The largest observed bottleneck for each preferred tunnel is shown for all simulations; the pseudo-apo and true-apo tunnels are shown in black, dots and lines, respectively, and the pseudo-holo and true-holo simulations are shown in grey with the same symbols.
(TIF)
