# Supplementary figures and images for: Ensemble Generation and the Influence of Protein Flexibility on Geometric Tunnel Prediction in Cytochrome P450 Enzymes
Source: PLoS One. 2014 Jun 23;9(6):e99408. doi: 10.1371/journal.pone.0099408 (PMC4067289; doi:10.1371/journal.pone.0099408)

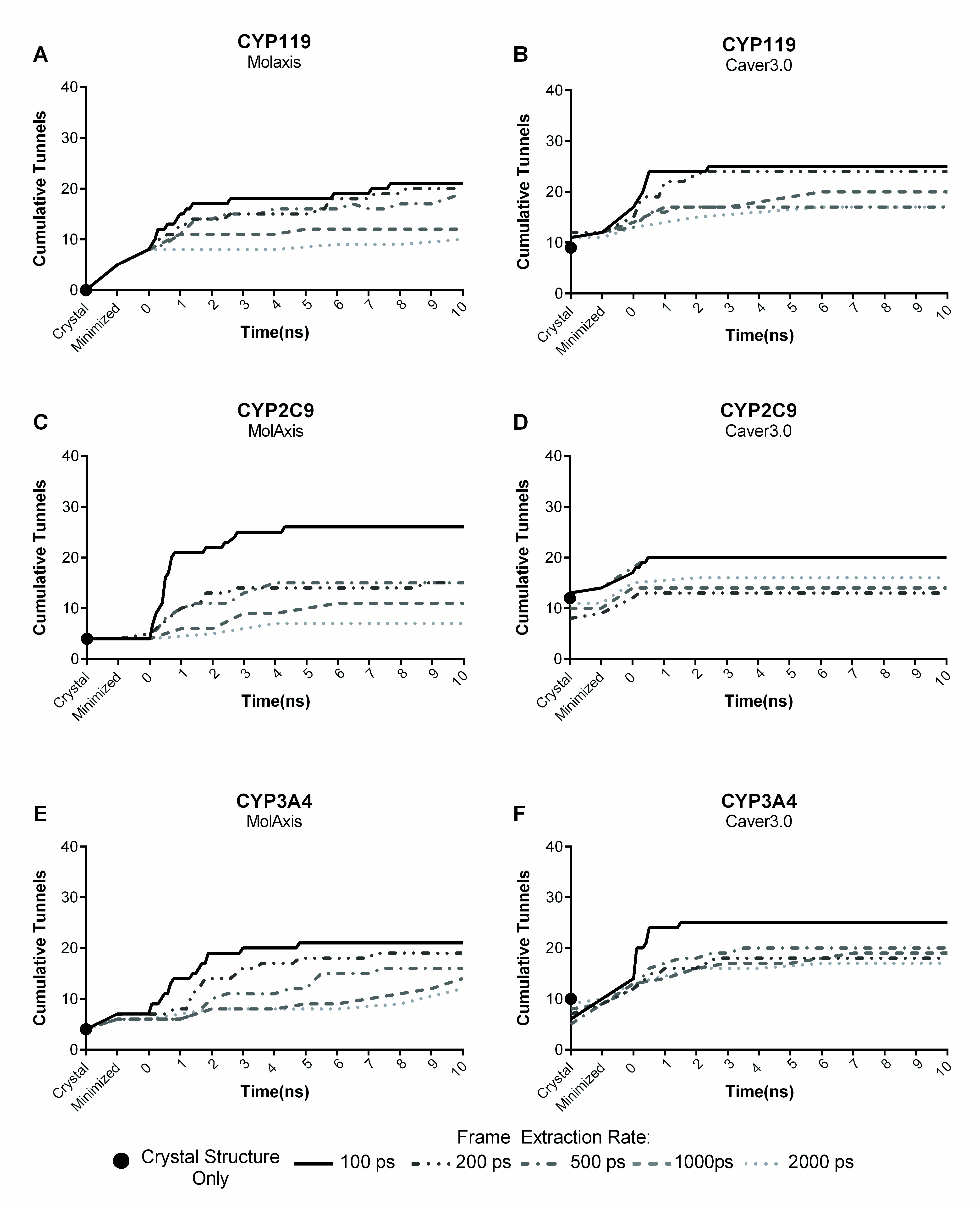

Supplement: Figure S1 — Cumulative number of tunnels found over time in the apo forms of CYP119 (A and B), CYP2C9 (C and D), and CYP3A4 (E and F) using the geometric prediction programs MolAxis (left column) and Caver3.0 (right column). Tunnels were predicted in five different ensembles, containing either 103, 53, 23, 13, or 8 members, generated by taking frames at evenly separated time points throughout the trajectory and adding the crystal and minimized structures. The tunnels were then clustered and the first appearance of each tunnel cluster was recorded. The black dot represents the number of tunnels in the crystal structure alone. (TIF) [file pone.0099408.s001.tif]

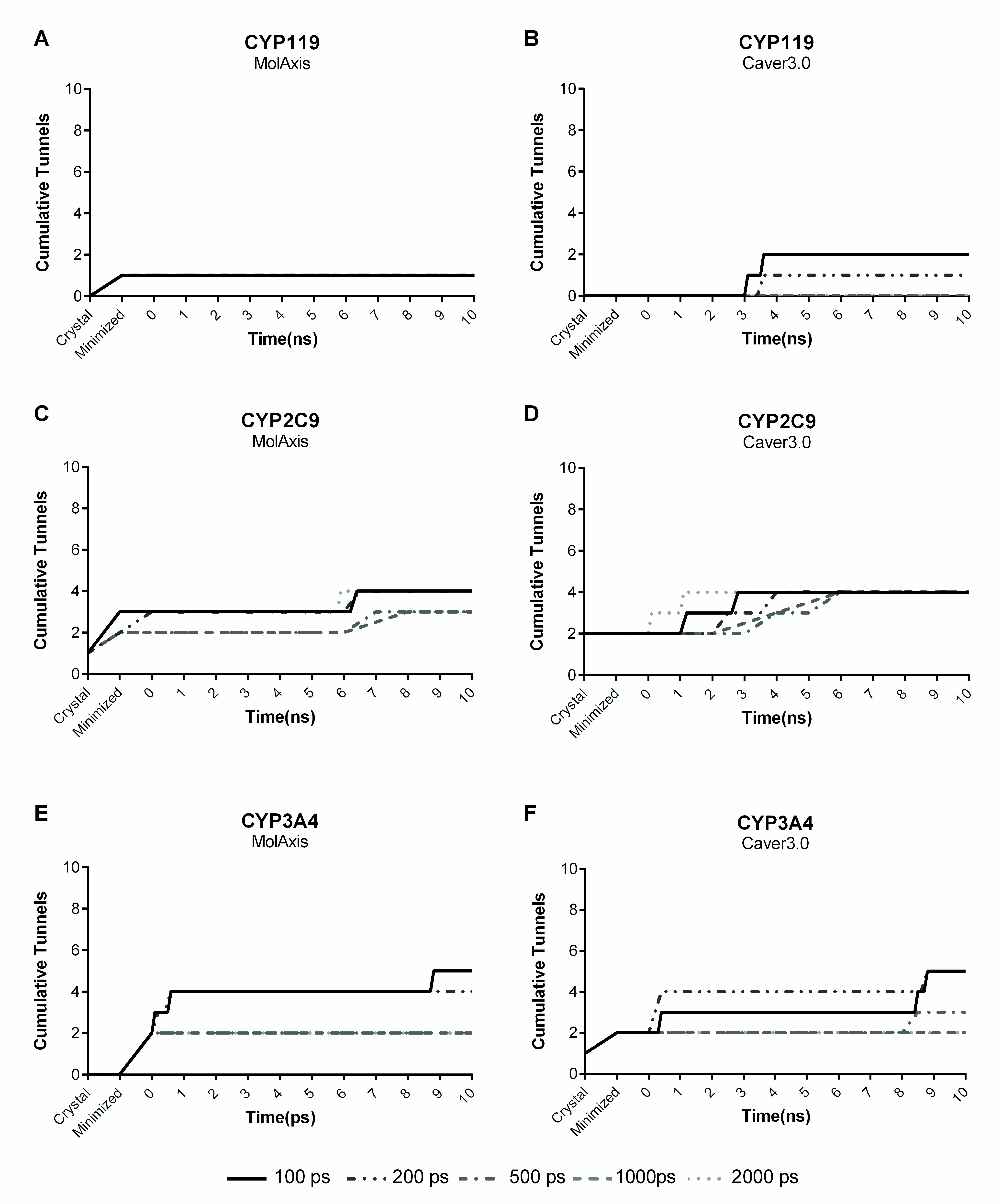

Supplement: Figure S2 — Tunnel prediction as a function of sampling time and ensemble size in the apo ensembles (1.25 Å cutoff). Cumulative number of tunnels found over time using a bottleneck cutoff of 1.25 Å in the apo forms of CYP119 (A and B), CYP2C9 (C and D), and CYP3A4 (E and F) using the static prediction programs, MolAxis (left column) and Caver3.0 (right column). Tunnels were predicted in five different ensembles, containing either 103, 53, 23, 13, or 8 members, which were generated by taking frames at specific time points throughout the trajectory and adding the crystal and minimized structures. (TIF) [file pone.0099408.s002.tif]

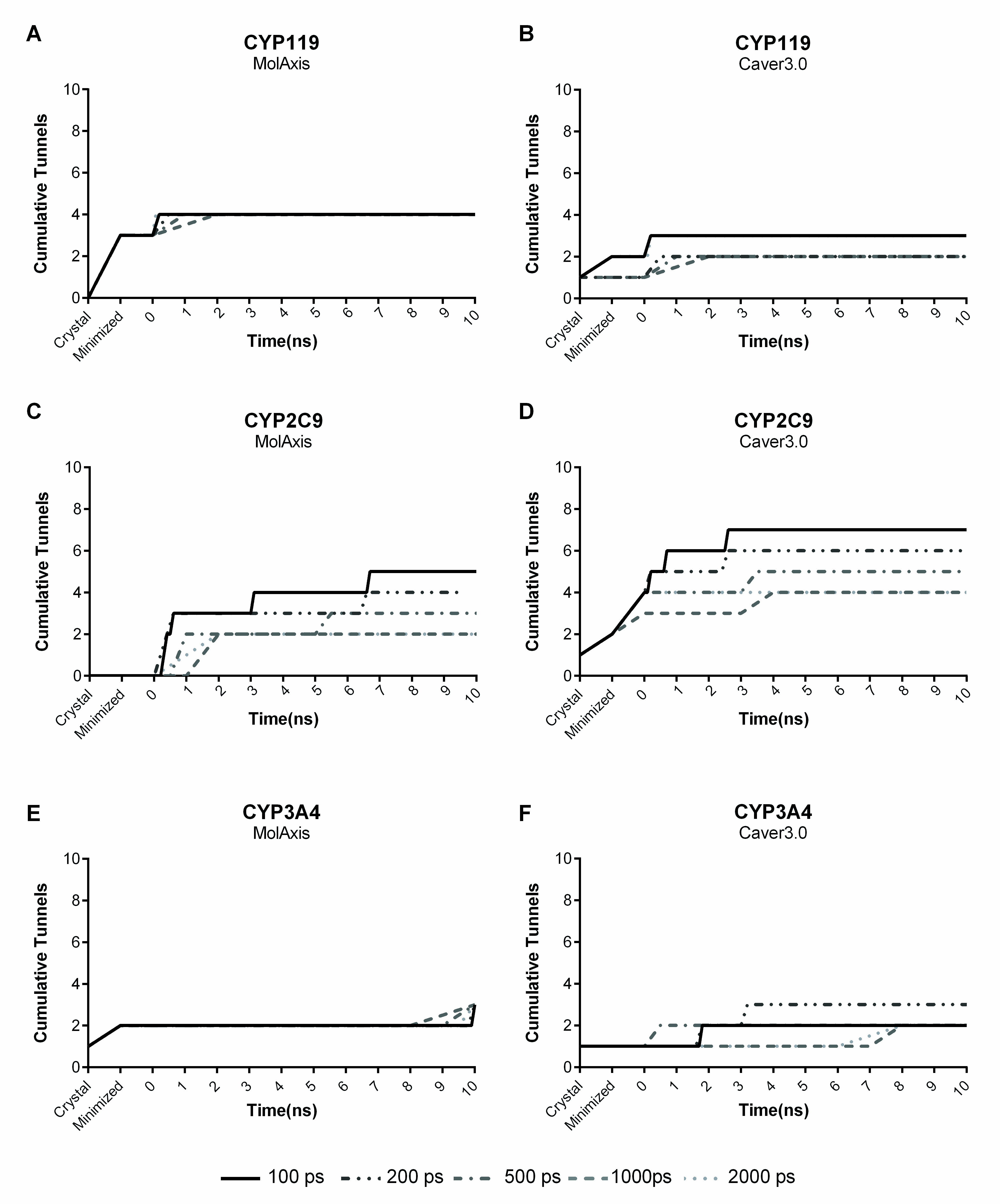

Supplement: Figure S3 — Tunnel prediction as a function of sampling time and ensemble size in the holo ensembles (1.25 Å cutoff). Cumulative number of tunnels found over time using a bottleneck cutoff of 1.25 Å in the holo forms of CYP 119 (A and B), CYP2C9 (C and D), and CYP3A4 (E and F) using the static prediction programs, MolAxis (left column) and Caver3.0 (right column). Tunnels were predicted in five different ensembles, containing either 102, 52, 22, 12, or 7 members, which were generated by taking frames at specific time points throughout the trajectory and adding the crystal and minimized structures. (TIF) [file pone.0099408.s003.tif]

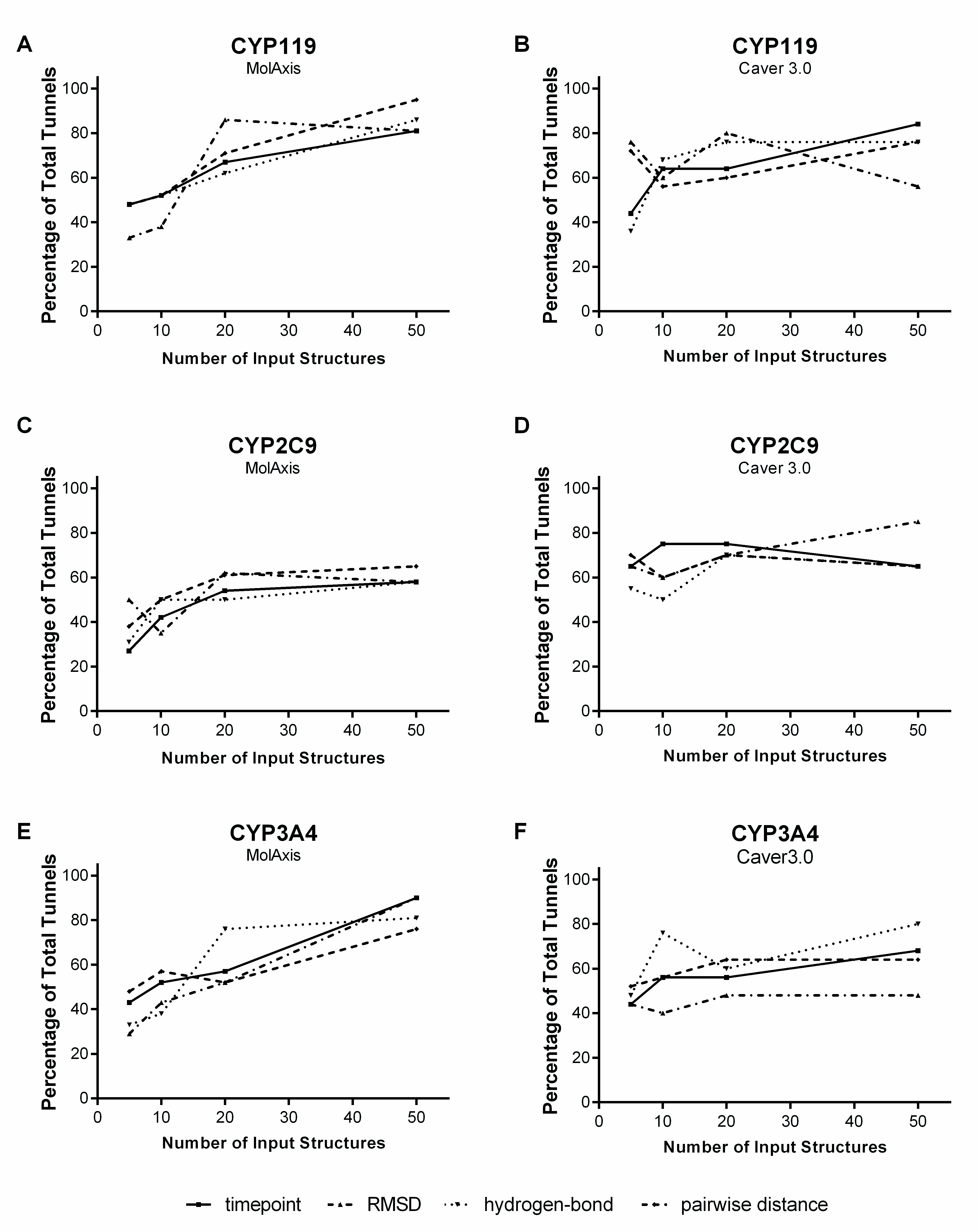

Supplement: Figure S4 — Percentage of the total reference tunnels identified using time point ensembles, RMSD-clustered ensembles, hydrogen-bond network ensembles, and pairwise-distance ensembles in the holo form of CYP119 (A and B), CYP2C9 (C and D) and CYP3A4 (E and F) using MolAxis and Caver3.0. (TIF) [file pone.0099408.s004.tif]

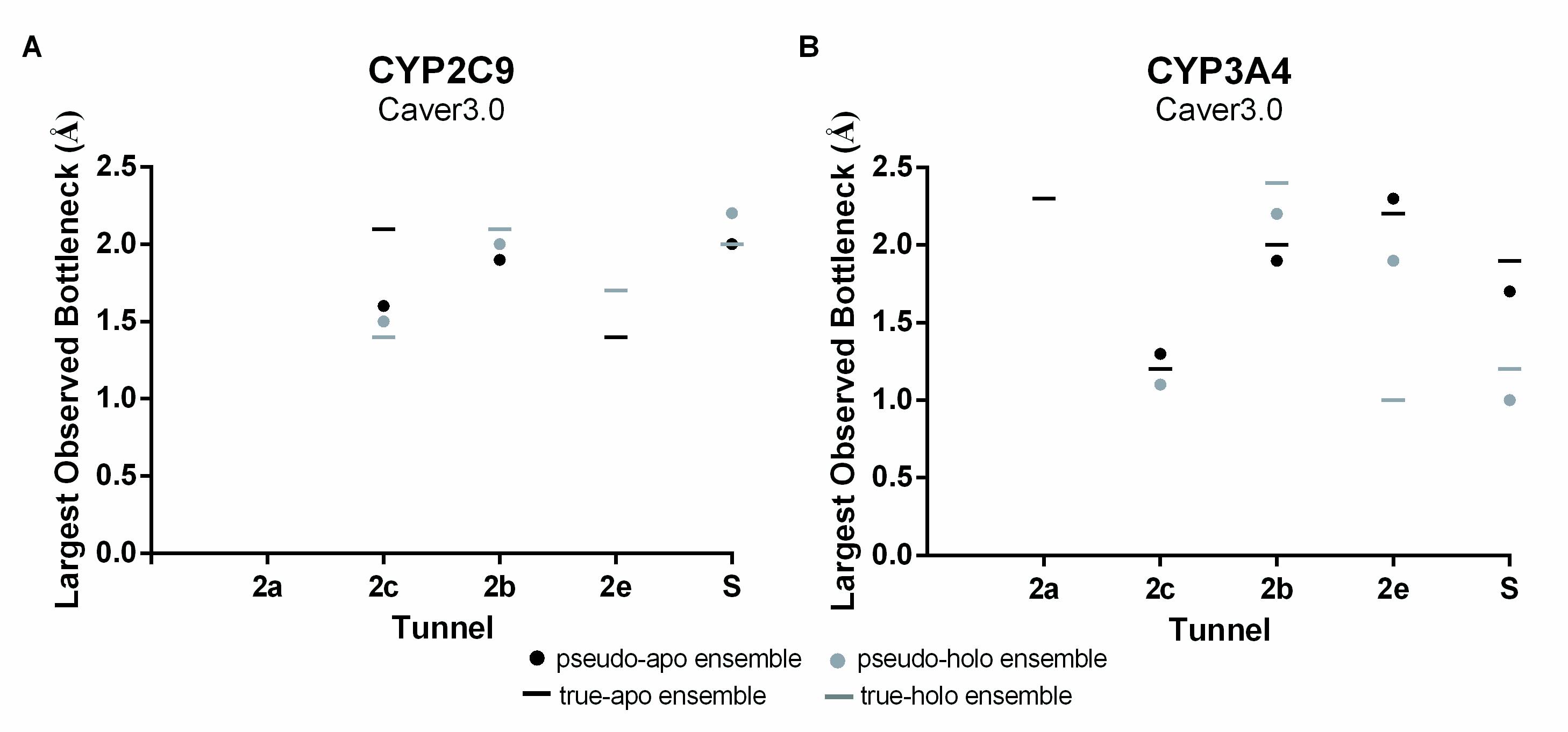

Supplement: Figure S5 — A comparison of the bottleneck radii in the preferred tunnels reported by Caver3.0 in the true- and pseudo- ensembles of CYP2C9 (A) and CYP3A4 (B). The largest observed bottleneck for each preferred tunnel is shown for all simulations; the pseudo-apo and true-apo tunnels are shown in black, dots and lines, respectively, and the pseudo-holo and true-holo simulations are shown in grey with the same symbols. (TIF) [file pone.0099408.s005.tif]
